# Anisotropic proximity–induced superconductivity and edge supercurrent in Kagome metal, K_1−*x*_V_3_Sb_5_

**DOI:** 10.1126/sciadv.adg7269

**Published:** 2023-07-12

**Authors:** Yaojia Wang, Shuo-Ying Yang, Pranava K. Sivakumar, Brenden R. Ortiz, Samuel M. L. Teicher, Heng Wu, Abhay K. Srivastava, Chirag Garg, Defa Liu, Stuart S. P. Parkin, Eric S. Toberer, Tyrel McQueen, Stephen D. Wilson, Mazhar N. Ali

**Affiliations:** ^1^ Max Planck Institute of Microstructure Physics, 06108 Halle, Saxony-Anhalt, Germany.; ^2^ Kavli Institute of Nanoscience, Delft University of Technology, Delft, The Netherlands.; ^3^ Materials Department, University of California Santa Barbara, Santa Barbara, CA 93106, USA.; ^4^ IBM Almaden Research Center, San Jose, CA 95120, USA.; ^5^ Department of Physics, Beijing Normal University, Beijing 100875, China.; ^6^ Colorado School of Mines, Goldon, CO 80401, USA.; ^7^ Johns Hopkins University, Baltimore, MD 21218, USA.

## Abstract

Materials with Kagome nets are of particular importance for their potential combination of strong correlation, exotic magnetism, and electronic topology. KV_3_Sb_5_ was discovered to be a layered topological metal with a Kagome net of vanadium. Here, we fabricated Josephson Junctions of K_1−*x*_V_3_Sb_5_ and induced superconductivity over long junction lengths. Through magnetoresistance and current versus phase measurements, we observed a magnetic field sweeping direction–dependent magnetoresistance and an anisotropic interference pattern with a Fraunhofer pattern for in-plane magnetic field but a suppression of critical current for out-of-plane magnetic field. These results indicate an anisotropic internal magnetic field in K_1−*x*_V_3_Sb_5_ that influences the superconducting coupling in the junction, possibly giving rise to spin-triplet superconductivity. In addition, the observation of long-lived fast oscillations shows evidence of spatially localized conducting channels arising from edge states. These observations pave the way for studying unconventional superconductivity and Josephson device based on Kagome metals with electron correlation and topology.

## Teaser

K1-xV3Sb5 Josephson junctions show internal magnetic field, edge supercurrent, and possible unconventional superconductivity.

## INTRODUCTION

The Kagome lattice, formed by corner-sharing triangles of atoms, is an important structure type that is proximate to the honeycomb (like graphene), hosting topological band structures, electron correlation, and geometrical frustration (*1*–*3*). It is an ideal platform for investigating many exotic electronic behaviors such as the quantum spin liquid state, unconventional superconductivity, and Dirac/Weyl/Nodal line semimetal behavior (*3*–*6*). Recently, the family of Kagome metals AV_3_Sb_5_ (A = K, Cs, and Rb) (*7*) has attracted substantial attention due to the observation of diverse physical properties including charge-density waves (CDWs) (*8*–*13*), superconductivity (*14*–*17*), a giant anomalous Hall effect (AHE) (*18*, *19*), and topological states (*14*, *18*, *20*, *21*).

Many studies have been performed to explore and understand the nature of these properties and electronic states in the AV_3_Sb_5_ quantum material family. While no long-range magnetic order has been observed through inelastic neutron scattering (*7*), muon spin relaxation experiments have shown signals of time-reversal symmetry (TRS) breaking with weak internal local magnetic field below CDW transition in KV_3_Sb_5_ and CsV_3_Sb_5_ (*22*–*24*). This aligned well with the observed giant extrinsic skew scattering AHE below the CDW transition (*18*, *19*). In addition, chiral charge ordering with magnetic field tunability and charge order states breaking rotation symmetry were detected in CsV_3_Sb_5_ and KV_3_Sb_5_ (*9*, *11*, *12*, *25*–*27*), and possible unconventional superconductivity in AV_3_Sb_5_ was also discussed (*11*, *12*, *22*, *28*). Theoretically, several ground states of AV_3_Sb_5_ were proposed including a star-of-David structure, tri-hexagonal structures, and TRS breaking states such as a chiral flux state, charge/spin bond orders, spin density waves, and excitonic order (*29*–*35*). However, differing reports on the presence or absence of these order states and the nature of the superconductivity (*25*, *36*–*38*) has made understanding the magnetism and superconductivity in the AV_3_Sb_5_ family difficult, and there is a lot of space to excavate the underlying physics.

In addition to looking for intrinsic superconductivity, using the proximity effect is another route to investigate the superconducting and electronic properties of materials. The quantum material Josephson junction (JJ), where two superconductors (SC) are coupled via a bridge of nonsuperconducting (non-SC) quantum material, is an effective structure to induce superconductivity. In a JJ, proximity-induced Cooper pairs are very sensitive to the barrier properties, and the resultant interference effects based on the coupling of the SC wave functions can reveal inherent physical properties of the barriers, including topological states and magnetism (*39*–*41*). On the other hand, the JJ is an important structure to explore intriguing physical properties, such as generating spin-triplet Cooper pairs in JJs of magnetic barriers (*42*–*49*) and exploring possible topological superconductivity through superconductivity of a topological state (*50*, *51*). Thus, JJs with topological Kagome metal barriers are a great tool to explore intriguing superconducting properties and understand the physical properties of Kagome materials.

In this work, we fabricated JJs of intrinsically non-SC, potassium-deficient K_1−*x*_V_3_Sb_5_ (*x* ~ 0.26 to 0.31) and then induced superconductivity through proximity. The Josephson effect is observed in long channels up to 6 μm, and the JJs exhibit a prominent asymmetry and reversion of the magnetoresistance for the up and down magnetic field sweeps only in the superconducting state of the JJ. Moreover, the interference pattern of critical current (*I*_c_) versus magnetic field shows a typical Fraunhofer-like pattern for an applied in-plane field but an anomalous pattern with a minimum near-zero field for an applied out-of-plane field. These unusual magnetic field modulations of supercurrent with strong directional anisotropy indicate that the superconducting coupling in the junction is influenced by the internal anisotropy of K_1−*x*_V_3_Sb_5_, which should be related to the charge order state and the internal magnetic field. The possible spin-triplet pairing in the JJs is discussed. Last, a nonvanishing, fast oscillation of *I*_c_ with a scalloped peak profile and excitation branches was observed, indicating the presence of edge localized supercurrent. Theoretical analysis of the band structure confirms the expectation of topological surface states in the (010)/(100) plane of KV_3_Sb_5_, corresponding to the edges of our samples. This solidifies K_1−*x*_V_3_Sb_5_ as an exciting platform to study the interplay of superconductivity, unconventional charge order state, and topology and also to show the potential of Kagome materials JJ for exploring unconventional superconductivity.

## RESULTS

### Proximity effect–induced superconductivity

**Fig. 1.**
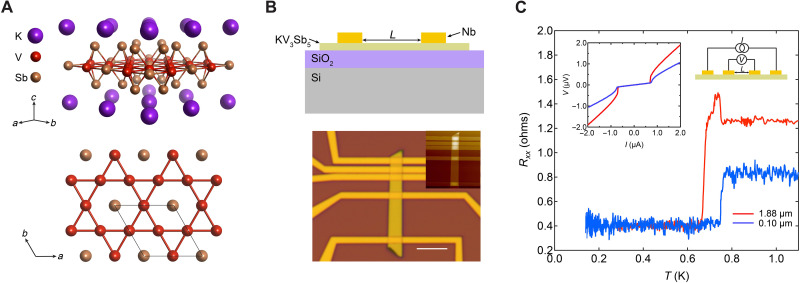
Crystal structure and JJ of KV_3_Sb_5_. (**A**) Top: Crystal structure of KV_3_Sb_5_. Bottom: Projection along the *c* axis showing the Kagome net of vanadium. (**B**) Top: Side-view schematic of K_1−*x*_V_3_Sb_5_ JJ. Bottom: The optical and atomic force microscopy (inset) image of one fabricated JJ device (device #1) of K_1−*x*_V_3_Sb_5_ thin flake (~ 45 nm). Scale bar, 5 μm. (**C**) Superconducting transition of device #1 measured by four-probe method. The insets are schematic of measurement circuit (top right) and typical voltage versus current curves measured at 20 mK (top left).

KV_3_Sb_5_ crystallizes in the hexagonal centrosymmetric space group *P*6/mmm and is composed of Kagome layers of vanadium interleaved with honeycomb layers of Sb and K (Fig. 1A). JJs with different channel lengths were fabricated on K_1−*x*_V_3_Sb_5_ nanoflakes with Nb [critical temperature (*T*_c_) ~ 6 K] as the superconducting electrodes. The nanoflakes were exfoliated from the same batch of crystals used in the work of Yang *et al.* (*18*), which were grown using the same method as previously described by Ortiz *et al.* (*7*). It should be noted that, although stoichiometric KV_3_Sb_5_ can intrinsically superconduct (*15*), the potassium-deficient K_1−*x*_V_3_Sb_5_ flakes can be non-SC. We fabricated K_1−*x*_V_3_Sb_5_ devices with Au contacts to study the intrinsic properties at ultralow temperatures. Superconductivity was observed in some Au devices with *T*_c_ ~ 0.6 to 0.65 K and *I*_c_ ~ 13 to 18 μA but was absent in others. Using electron-dispersive x-ray spectroscopy measurements, it was revealed that there were different compositions in the superconducting Au-contacted samples compared with the non-SC samples; the superconducting samples were closer to the ideal 1:3:5 stoichiometry compared to the non-SC samples (see details in section S3), which is consistent with the observation of intrinsic superconductivity in stoichiometric bulk KV_3_Sb_5_ with a maximum *T*_c_ ~ 0.93 K (*15*). The reduced *T*_c_ in the deficient samples compared with stoichiometric KV_3_Sb_5_ and the disappearance of superconductivity in our K_1−*x*_V_3_Sb_5_ samples with low-potassium concentration indicate that the superconductivity in KV_3_Sb_5_ is highly dependent on defect/doping. In this work, the non-SC K_1−*x*_V_3_Sb_5_ samples with highly potassium deficiency (*x* = 0.26 to 0.31) were used to fabricate the JJs.

Figure 1B shows a schematic structure of a Nb/K_1−*x*_V_3_Sb_5_/Nb device and one of the fabricated devices with channel length *L* varying from 0.1 to 6.05 μm with a constant sample thickness of ~45 nm (device #1). We first measured *R* versus *T* curves of both short (*L* = 0.1 μm) and intermediate channels (*L* = 1.88 μm) by the four-probe method to exclude the contact resistance (results shown in Fig. 1C). A sudden drop in resistance is observed at temperatures of 0.76 and 0.68 K for channels of *L* = 0.1 and 1.88 μm, respectively, with the same artifactual residual resistance value from lock-in measurements (see discussion in section S1). The *I* versus *V* curves measured at ~20 mK show a sudden jump in voltage above *I*_c_, further confirming the superconducting state at low temperature (Fig. 1C, inset).

**Fig. 2.**
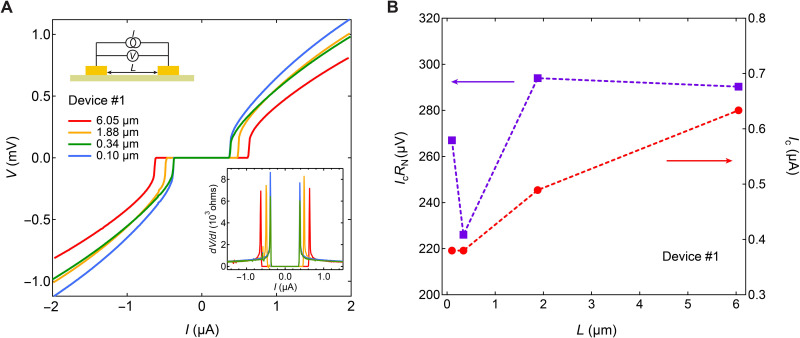
Length dependence of Josephson current. (**A**) Voltage versus current (*V*-*I*) curves for different channel lengths measured by two-probe method at 20 mK. The insets are corresponding differential resistance (down right) and schematic of measurement circuit (top left). (**B**) Length dependent *I*_c_ and *I*_c_*R*_N_ extracted from the *V*-*I* curves in (A).

To verify that the superconducting signal is related to the proximity effect from the superconducting electrodes, we investigated the length dependence of superconductivity by measuring the *I* versus *V* curves of different channel lengths at 20 mK using the two-probe method. Figure 2A shows the results measured in device #1; the superconducting transition is observed up to 6.05 μm. The extracted *I*_c_ (taken from the peak location on *dV*/*dI* versus *I* curve) is small (*I*_c_ < 1 μA) and different for different channel lengths, consistent with proximity-induced superconductivity. Unexpectedly, *I*_c_ increases with increasing channel length (Fig. 2B, red dashed line), distinct from a typical JJ with a transparent interface in which *I*_c_ monotonically reduces with increasing channel length (at fixed thickness and width) due to reduced coupling between the two superconducting electrodes (*52*). However, because the contact resistance of the interface is higher than the sample resistance in our device, the interface of each channel needs to be considered. We extracted the *I*_c_*R*_N_ of different JJs, where *R*_N_ is the normal state resistance of the junction. As shown in Fig. 2B (purple dashed line), *I*_c_*R*_N_ varies weakly with channel length, indicating that the *I*_c_ is being dominated by the interface of the SC with the K_1−*x*_V_3_Sb_5_ barrier; similar *I*_c_*R*_N_ are also observed in other devices (see results of device #2 in section S2). This again shows that the supercurrent is induced by the proximity effect of the superconducting electrodes.

**Fig. 3.**
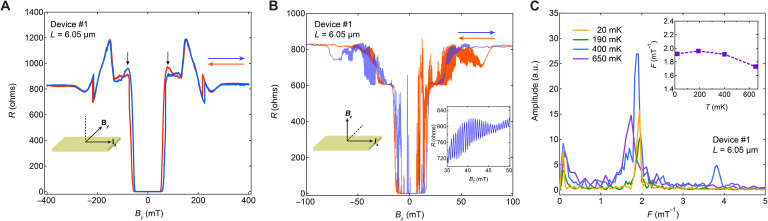
Magnetic field–dependent resistance of JJ with *L* = 6.05 μm. (**A**) *R* versus *B* with magnetic field applied in-plane but orthogonal to the current. Red (blue) line and arrows denote field downsweeping (upsweeping). The two black arrows point out the reversion of resistance for different sweep directions. The inset denotes magnetic field and current directions. (**B**) *R* versus *B* of the same device with magnetic field applied out of plane and orthogonal to the current. The left inset denotes magnetic field and current directions, and the right inset is the enlarged plot of fast oscillation. (**C**) Fast Fourier transform of fast oscillation in (B) at different temperatures; the inset is temperature-dependent oscillation frequency obtained in the main panel of (C). a.u., arbitrary units.

**Fig. 4.**
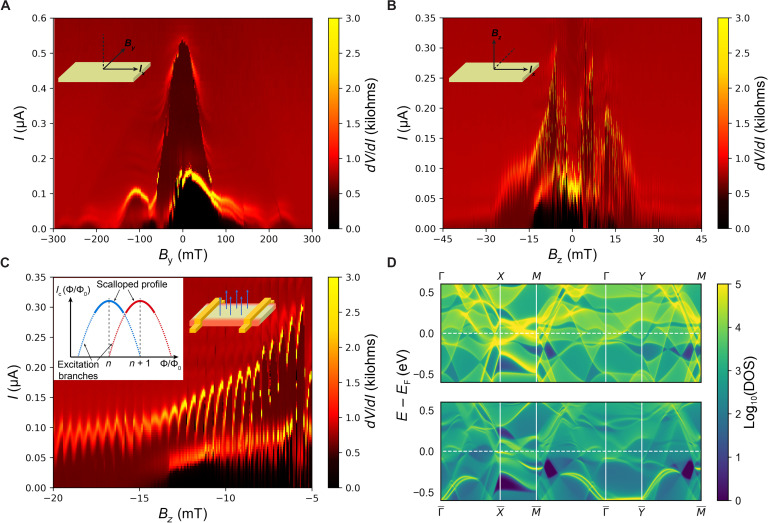
Interference patterns and localized Josephson current. (**A** and **B**) Color maps of *dV*/*dI* versus current and magnetic field measured in device #1 at 20 mK when applying in-plane magnetic field and out-of-plane magnetic field, respectively. The field is changing from positive to negative during the measurement. The insets denote magnetic field and current directions. (**C**) Main panel: The enlarged plots of the fast oscillation in (B) when applying out-of-plane field. The right inset is a schematic of the edge supercurrent in the JJ, the edges are indicated by red color. The left inset is a schematic current profile of an edge state. (**D**) Top: Bulk spectral density of states (DOS) for KV_3_Sb_5_ on the (010)/(100) surfaces. Bottom: Surface spectral density of states for the (010)/(100) surfaces. Several bright bands are present in the bottom panel but not in the top panel; these are the surface states.

The proximity effect–induced superconductivity is also supported by many other pieces of evidence. The superconducting transition temperatures and *I*_c_ of different junctions made on the same flake (with uniform width and thickness) are different (Figs. 1C and 2), which is fundamentally distinct from an intrinsically superconducting flake with uniform width and thickness that would have constant *T*_c_ and *I*_c_. In addition, we compare the magnetic field dependence of the resistance and *I*_c_ (discussed in detail later) in highly potassium-deficient Nb/K_1−*x*_V_3_Sb_5_/Nb junction (Figs. 3 and 4) with Au-contacted superconducting K_1−*x*_V_3_Sb_5_ sample (fig. S4). A Fraunhofer pattern was observed (Fig. 4A) in the Nb-contacted junction but disappeared in the Au-contacted superconducting sample (fig. S4), which is a strong evidence for Josephson coupling in our Nb/K_1−*x*_V_3_Sb_5_/Nb junction, and against intrinsic superconductivity. Moreover, the critical field (*B*_c_) of superconductivity in the Nb-contacted junction (e.g., *B*_c_ ~ 300 mT for in-plane field and ~ 85 mT for out-of-plane field for *L* = 6.05 μm) is much higher than that in the intrinsic Au-contacted superconducting K_1−*x*_V_3_Sb_5_ (~100 mT for in-plane field, and ~15 to 20 mT for out-of-plane field). The Fraunhofer pattern also sustains up to ~300 mT (Fig. 4A) for in-plane field. These results strongly indicate that the superconductivity in Nb-contacted junctions is coming from the proximity effect of superconducting electrodes. In addition, the contribution of inhomogeneous superconductivity in the junction can also be excluded. A more comprehensive discussion of the proximity effect–induced superconductivity is included in section S4.

While the proximity length up to 6.05 μm is quite long, much longer than the coherence length (*ξ_N_*~230 nm) calculated on the basis of bulk mobility of the barrier material (see details in Methods), similar results have been observed in JJs with a giant proximity effect when the non-SC bridge material in a JJ is close to superconducting itself (i.e., can superconduct with mild doping or temperature is above *T*_c_ of barrier) (*53*, *54*) or in topological materials with topological edge/surface states (*55*, *56*). Because stoichiometric KV_3_Sb_5_ can be an intrinsic SC, the K_1−*x*_V_3_Sb_5_ sample in the JJ may be relatively near a superconducting state and helping create the long Josephson length. This may also be the reason of weaker length dependence of superconductivity in our junction compared with typical JJs. In addition, the topological properties of K_1−*x*_V_3_Sb_5_ (addressed below) may also contribute to the observed long junction lengths.

### Magnetic field sweep direction dependence of magnetoresistance

We next study the magnetic field dependence of superconductivity in the K_1−*x*_V_3_Sb_5_ JJs with both in-plane and out-of-plane magnetic field. Figure 3 shows the results of the channel with *L =* 6.05 μm in device #1. When applying the magnetic field (labeled *B_y_*) in the sample plane perpendicular to the current direction (labeled *I_x_*) at 20 mK, the breaking of superconductivity is observed at an in-plane *B*_c_ of ~300 mT (Fig. 3A). In particular, the *R* versus *B* curve is asymmetric about zero field for positive and negative field, but the curve reverses for upsweep (blue line) and downsweep (red line) of the field, as indicated by the black arrows in Fig. 3A. This property is also observed when applying an out-of-plane magnetic field (*B_z_* direction). As shown in Fig. 3B, the *R* versus *B* curves at 20 mK present a prominent asymmetry and reversion for upsweep and downsweep with an out-of-plane *B*_c_ of ~85 mT. This flipping property of the *R* versus *B* curves is observed in all the measured K_1−*x*_V_3_Sb_5_ JJs and channels for both in-plane and out-of-plane fields (see more data for other channels in fig. S6). Moreover, Fig. 3B presents a prominent fast oscillation overlaid on the background of the *R* versus *B*. Using fast Fourier transform analysis, the frequency of fast oscillation is extracted and found to be 1.9 mT^−1^ and is not sensitive to temperature until very near the superconducting transition, as shown in Fig. 3C (additional data in fig. S9). This robust fast oscillation is related to the interference of supercurrent and is modulated by the magnetic flux, like a superconducting quantum interference device (SQUID), and its origin is discussed in detail below. Similar to the background of the *R* versus *B* curves, the fast oscillation also reverses for different field sweep directions (Fig. 3B).

### Anisotropic interference pattern for in-plane and out-of-plane magnetic field

To further understand the magnetic field dependence of superconductivity, the magnetic field modulation of the critical supercurrent in the K_1−*x*_V_3_Sb_5_ JJs is studied. Figure 4 (A and B) is the color maps of *dV*/*dI* versus *I* and *B* for in-plane field and out-of-plane field, respectively, measured by downsweep of the field for the 6.05-μm JJ (see fig. S8 in for upsweep pattern). There are two sets of interference patterns (bright lines) on the color maps, which may be indicative of distinct superconducting channels in the K_1−*x*_V_3_Sb_5_ JJ (see detailed discussion in section S8). Because the outside pattern sustains to higher field than the inside pattern and dominates the primary interference features, we focus on the outside pattern. When applying the in-plane magnetic field, with *B_y_*⊥*I_x_*, the main interference pattern of *I*_c_ versus *B* shows a prominent peak at zero field, and the oscillation of *I*_c_ decays quickly with increasing field, as is expected of a Fraunhofer-like *I*_c_ versus *B* pattern for a JJ. This confirms that Josephson coupling is achieved in these devices. However, when applying an out-of-plane (*B_z_*) magnetic field (Fig. 4B), the interference pattern shows an anomalous suppression near-zero field, resulting in a minimum instead of a peak, at the center of the interference pattern. Under a weak magnetic field (either up or down), the *I*_c_ is enhanced relative to zero field but is still smaller than the maximum *I*_c_ with in-plane field. This anisotropic interference pattern and suppression of *I*_c_ are quite unusual. In addition, a long-lived fast, SQUID-like oscillation is also clearly visible on top of the primary background in Fig. 4B. Because the superconductivity presents many properties, we discuss these properties piecewise and their related physical origins in detail below.

## DISCUSSION

### Anisotropic internal magnetic field and spin-triplet superconductivity

The results in Figs. 3 and 4 show the distinct magnetic field dependence of superconductivity in a K_1−*x*_V_3_Sb_5_ JJ including the reversion of the *R* versus *B* curves when changing field sweeping direction and anisotropic interference patterns with central peak for in-plane field but abnormal suppression of *I*_c_ for out-of-plane field. These features are also observed in the superconducting state of other Nb-contacted K_1−*x*_V_3_Sb_5_ junctions (figs. S5 and S6) but disappear in Au-contacted superconducting devices (see details in fig. S4), indicating that they are properties of the proximity-induced supercurrent in the JJs.

In KV_3_Sb_5_, no long-range magnetic order has been observed down to 0.25 K via neutron scattering (below the *T*_c_ of these JJs) (*7*); however, it has presented large AHE, and a signal of TRS breaking with weak internal local magnetic field was detected below the CDW transition via muon spin relaxation (*22*); similar TRS breaking below CDW is also widely observed in its sister materials CsV_3_Sb_5_ (*23*, *57*) and RbV_3_Sb_5_ (*58*). We note that the signal of CDW transition and AHE also appear in our non-JJ, Au-contacted non-SC K_1−*x*_V_3_Sb_5_ samples (see data in fig. S3), which indicates that the TRS breaking with internal magnetic field is preserved in our potassium-deficient samples. In JJs, the internal magnetic field of the barrier will influence the injection and transport of Cooper pairs (*42*), which can lead to the reversion of *R* versus *B* curves, as has been widely observed in JJs with barrier materials breaking TRS, such as ferromagnets (*59*) and magnetic element–doped barriers (*49*). The reversion of magnetoresistance is robustly observed in all K_1−*x*_V_3_Sb_5_ JJs with different channel lengths, indicating that it is an intrinsic property that should be induced by the internal local magnetic field in K_1−*x*_V_3_Sb_5_. As a control, we also fabricated the Nb/graphene/Nb JJ, and the reversion feature is absent, as expected (see data of a Nb/graphene/Nb JJ in fig. S7).

The distinct interference patterns for in-plane and out-of-plane field in K_1−*x*_V_3_Sb_5_ JJ imply that the internal field is also anisotropic, as the superconducting coupling in the junction was modulated differently when changing the direction of the external magnetic field. Recently, muon spin relaxation experiments have reported anisotropy of the internal local magnetic field between in-plane and out-of-plane directions in the sister compound CsV_3_Sb_5_ (*23*). The emergence of local internal field was found to be associated with the charge order transition (*23*, *24*). So far, the low-temperature order states in KV_3_Sb_5_ and CsV_3_Sb_5_ are still not clear, with several types of charge/spin orders breaking TRS having been proposed, including a chiral flux phase (*32*), charge/spin bond order (*29*, *34*), and spin density wave (*31*). Note that the easy plane of these orders lies in-plane (Kagome lattice), and it was also proposed that the out-of-plane magnetic field (along the *c* axis) can modulate these orders (*31*), and obvious enhancement of the magnetic field signal under out-of-plane field was detected in muon and optical experiments (*23*, *57*). The field modulatable ordered states and related internal magnetic field likely contribute to the anisotropic magnetic field response of supercurrent in the JJ.

Associated with this anisotropic magnetic field dependence of the supercurrent, we discuss the origin of the suppressed *I*_c_ with a minimum near-zero field in the interference pattern (out-of-plane field). In general, a JJ with normal spin-singlet pairing of nonmagnetic barriers displays a standard single-slit Fraunhofer interference pattern, with periodic oscillations and a central maximum. The unusual central minimum of *I*_c_ normally appears in JJs with magnetic barriers where the junction has modulated phase and spin-triplet paring (*42*, *60*). A very similar suppression and minimum of *I*_c_ was observed in JJs with long range spin-triplet pairing, such as the JJ of half-metal CrO_2_ and in hybrid superconductor/ferromagnet junctions (*45*, *61*). The conversion of spin singlet to triplet pairing in JJs generally happens on the basis of inhomogeneous magnetizations, noncollinear spin structures, or spin spiral structures, which breaks spin rotation symmetry allowing spin-flipping at the interfaces (*42*, *44*, *47*).

For the KV_3_Sb_5_ family, as discussed before, TRS breaking was reported in many works, and several possible charge/spin order states were predicted in the Kagome plane (*23*, *29*, *31*, *32*, *34*, *57*), which can be modulated by external out-of-plane magnetic field (*23*, *31*, *57*), and many experiments have detected an enhanced magnetic field signal under this field direction (*23*, *57*). Among these, one widely discussed order is chiral charge order with loop current, which induces a varying local magnetic field (dependent on the hexagons versus triangles) in Kagome lattices (*24*, *32*, *57*). Other predicted spin orders also have varying spin directions (*29*, *31*, *62*). In addition, rotation symmetry breaking in KV_3_Sb_5_ (*25*) was also reported at low temperatures in scanning tunneling microscopy study. These broken symmetries combined with the spatial variation of local internal magnetic field make it possible to convert spin-singlet to spin-triplet supercurrent in K_1−*x*_V_3_Sb_5_ JJs and allow modulation of the superconducting coupling when the out-of-plane field tunes the ordered state and internal field. This also aligns with theoretical studies that found that both spin-singlet and spin-triplet pairing can appear in the KV_3_Sb_5_ family due to the complex band structure and electron correlations (particularly as a function of Fermi level that can be modulated by doping) (*28*, *63*, *64*) and that triplet pairing may be a favored state in the (relatively) weak correlation region (*28*). The combination of the observed abnormal interference pattern and these inherent properties of K_1−*x*_V_3_Sb_5_ indicates that spin-triplet pairing is possible in K_1−*x*_V_3_Sb_5_ JJs, and more experimental effort in future, including elucidation of the ground state in the AV_3_Sb_5_ family, will help understand the superconducting behavior in K_1−*x*_V_3_Sb_5_ JJs.

### Supercurrent of edge state

We next analyze the oscillations in the interference pattern and focus on the spatial distribution of the supercurrent and the prominent long-lived fast, SQUID-like oscillation of *I*_c_ in the Fig. 4. In the junction, the *I*_c_ is modulated by the magnetic flux in units of the flux quantum Φ_0_ with Φ_0_ = ∆*B·L·t*_eff_ (*L* ≈ 6.05 μm). On the basis of the Fraunhofer-like pattern (period of ∆*B* ~ 100 mT) for the in-plane magnetic field, an effective superconducting thickness of *t*_eff_ ≈ 3.4 nm is extracted. We also performed inverse Fourier transforms of the in-plane interference pattern (see section S9), and the obtained current density profile shows that the supercurrent is uniformly distributed through the top ~5 nm of the flake, which is in quite good agreement with *t*_eff_. These results imply that only a thin layer on the top of the flake is being proximitized, as expected; the superconducting order is not extending deeply along the *c* axis across the layers and is markedly longer in the *ab* plane. This thin proximitized layer is reasonable for K_1−*x*_V_3_Sb_5_ due to its layered structure, which is expected to be electronically anisotropic (the *c* axis versus the *ab* plane) like its sister material, CsV_3_Sb_5_, that has shown out-of-plane resistivity 600× larger than that in in-plane (*14*). This results in a small coherence length along the *c* axis, just like other layered materials including WTe_2_ (*65*).

The interference pattern for the out-of-plane magnetic field, however, with fast oscillations on top of the background is not a typical Fraunhofer pattern. The SQUID-like fast oscillation maintains a period of ∆*B*~0.5 mT that is consistent with the frequency of the oscillation on the *R* versus *B* curves and corresponds to an effective flux-penetration area of *S*_eff_ ~ 4.13 μm^2^ calculated from Φ_0_ = ∆*B*·*S*_eff_. This is smaller than the crystal area (*S* = *L*·*W* ≈ 12 μm^2^), which is likely caused by partial penetration of the flux, as was seen with MoTe_2_ (*66*). The most important feature of the fast oscillation is that it is nonvanishing and survives to higher field than the main interference pattern, as shown in Fig. 4B. This long-lived, fast oscillation cannot be induced by the bulk Josephson current. Moreover, it presents a scalloped peak profile with clear excitation branches trailing from the scalloped boundary, and prominent jumps between oscillation branches are observed (Fig. 4C). This scalloped profile and excitation branches of a nonvanishing fast mode are important signatures of nonbulk, spatially localized states (i.e., not uniformly distributed supercurrent) like surface or edge states (*40*, *66*, *67*). For an enclosed area bounded by the sides of the sample, the requirement of flux quantization results in a sawtooth profile of the surface Fermi velocity, which contributes to the scalloped profile and excitation branches, as observed in the edge state of MoTe_2_ (*66*). We also performed inverse Fraunhofer transform for the out-of-plane field interference pattern to extract the supercurrent density distribution along the width direction, and, although the quantitative analysis has some error due to the complicated *I*_c_ versus *B* pattern, the existence of two clear peaks in the current profile qualitatively indicates the presence of edge supercurrent (see details in fig. S11).

To examine the origin of this edge supercurrent, we further performed density functional theory (DFT) calculations of the electronic band structure of KV_3_Sb_5_. Previous works on both the isostructural and isoelectronic compounds CsV_3_Sb_5_ and KV_3_Sb_5_ have predicted ℤ_2_-protected topological surface Dirac crossings just above the Fermi level (*14*, *15*). Here, we calculated the spectral density of bulk (top panel) and surface states (bottom panel) particularly on the (100)/(010) planes (*ac*/*bc* planes), including states propagating along the [001] directions, as results shown in Fig. 4D. Although the large number of surface states prevents us from attributing the surface conductivity to a single surface band, our calculations show that there are prominent surface states near the Fermi level along the $\bar{X}-\bar{M}$ line, and the Fermi level of K_1−*x*_V_3_Sb_5_ [slightly below the predicted level, as shown by previous angle-resolved photoemission spectroscopy (ARPES) measurements (*18*)] cuts the surface states near the $\bar{X}$ point (corresponding to the edges in our thin flake JJ device). These surface states should contribute to the edge supercurrent shown as SQUID-like fast oscillations in the K_1−*x*_V_3_Sb_5_ JJ. See Methods and section S10 for more details of surface states. It should be noted that the surface states in the *ab* plane were not detected when applying in-plane field along the *y* axis, because only the top layers of the K_1−*x*_V_3_Sb_5_ samples were proximitized; coupling between the top and bottom surface states to result in the SQUID-like pattern was not possible, in analogy to what was seen with the hinge states in Al_2_O_3_ back-filled WTe_2_ devices (*68*). Detecting the *ab*-plane surface states is an area for future work and will require very thin flakes (sub–10 nm) or vertical JJ architectures.

In summary, we fabricated JJs of the topological Kagome metal, K_1−*x*_V_3_Sb_5_, and observed Josephson coupling through long distance; asymmetry and reversion of *R* versus *B* as well as a zero-field minimum in the *I*_c_ versus *B* pattern, which indicate anisotropic internal magnetism and possible spin-triplet superconductivity; and also the signal of supercurrent of the predicted topological surface states in K_1−*x*_V_3_Sb_5_. These observations in one system open the door for exploring emergent physical phenomena combination of superconductivity with unconventional charge order state and topology. Fundamentally, there is a variety of opportunities for future theoretical and experimental work to excavate unconventional superconductivity based on JJs of Kagome materials.

## METHODS

### Device fabrication and measurement

Thin flakes of KV_3_Sb_5_ were exfoliated onto an SiO_2_/Si substrate, followed by standard electron beam lithography and sputtering processes to fabricate the JJ devices with Niobium (~ 40 nm) as the superconducting electrodes. The JJs were measured in a Bluefors dilution refrigerator with base temperature reaching ~15 mK. The resistance of device was measured by applying different ac current excitations with low frequencies (below 30 Hz) and Zurich lock-in amplifiers. The differential resistance (*dV*/*dI*) was measured by adding an external dc source onto the small ac source of lock-in.

### DFT calculations

The electronic structure of KV_3_Sb_5_ was simulated in Vienna Ab-initio Simulation Package (VASP) v5.4.4 (*69*–*71*) using projector-augmented wave (PAW) potentials (*72*, *73*) with identical parameters to a previous work on CsV_3_Sb_5_ (*14*). Calculations used the Perdew-Burke-Ernzerhof (PBE) functional (*74*) with D3 dispersion corrections (*75*), a Γ-centered 11 × 11 × 5 *k*-mesh, a plane wave energy cutoff of 500 eV, and the recommended PAW potentials for v5.2. No magnetic moments were incorporated in the simulation. *a* and *c* lattice parameters of the relaxed cell were 5.42 and 8.92 Å, which are in good agreement with experimental lattice parameters of 5.48 and 8.95 Å, respectively. Similar PBE functional calculations have been shown to accurately recreate the slab electronic structure measured in ARPES experiments for both CsV_3_Sb_5_ (*14*) and KV_3_Sb_5_ (*18*). Wannier90 was used to generate an empirical tight-binding model starting from initial projectors corresponding to valence orbitals (*V p*, *d*; *Sb p*; with a frozen inner fitting window of Fermi energy *E*_F_ ± 2 eV and an outer window of *E*_F_ − 6 eV to *E*_F_ + 5 eV) (*76*). Surface density–of–state calculations were performed using the method of Sancho and co-workers (*77*) as implemented in WannierTools (*78*).

### Calculation of coherence length

For K_1−*x*_V_3_Sb_5_, according to equation μ = *el_e_*/ℏk_F_ with mobility μ~0.2 m^2^/*Vs* (*18*) and *k*_F_ ~ 0.032 Å^−1^, the mean free path of *l*_e_ ~ 42 nm is extracted. Because *l_e_* is smaller than the coherence length of Nb ($\xi_{S}=\frac{\hbar V_{F}}{1.76\pi k_{B}T_{c}}\approx$ 64 nm, *T*_c_ ≈ 6.2 K, *V*_F_ ≈ 3 × 10^5^ m/s) (*79*), the superconducting coherence length of spin-singlet pairing in nonmagnetic materials (*53*, *65*) or spin-triplet pairs in a magnetic system (*49*) can be calculated by $\xi_{N}=\sqrt{\hbar v_{F}l_{e}/6\pi k_{B}T_{c}}$ (Fermi velocity *v*_F_~3.77 × 10^5^ m/s, *T_c_* ~ 0.78 K), which is about 230 nm in K_1−*x*_V_3_Sb_5_.

## Supplementary Materials

### This PDF file includes:

Sections S1 to S10 Figs. S1 to S14
