# Supplementary material for: Anisotropic proximity–induced superconductivity and edge supercurrent in Kagome metal, K1−xV3Sb5
Source: Sci Adv. 2023 Jul 12;9(28):eadg7269. doi: 10.1126/sciadv.adg7269 (PMC10337911; doi:10.1126/sciadv.adg7269)
Supplement: Supplementary file 1 — Sections S1 to S10 Figs. S1 to S14 [file sciadv.adg7269_sm.pdf]

Science Advances
AAAS

Supplementary Materials for

Anisotropic proximity–induced superconductivity and edge supercurrent in Kagome metal, $K_{1-x}V_3Sb_5$

Yaojia Wang *et al.*

Corresponding author: Yaojia Wang, whyjwang@gmail.com; Mazhar N. Ali, maz@berkeley.edu

Sci. Adv. **9**, eadg7269 (2023)
DOI: 10.1126/sciadv.adg7269

This PDF file includes:

Sections S1 to S10
Figs. S1 to S14

S1. Residual resistance below the superconducting temperature

In our experiments, the measured resistance in all the devices is not zero below the superconducting temperature, resulting in a residual resistance. To examine whether the residual resistance is from an incomplete superconducting state in the $K_{1-x}V_3Sb_5$ JJs, we measured the superconducting transition in different instruments and compared different devices. We found that the measured resistance is dependent on the Lock-in equipment. Figure S1 shows different *R*-*T* curves of Device #1 with channel length L = 1.88 μm, which are measured by the same four-probe method using two different lock-in amplifiers. It is clear that two *R*-*T* curves show similar superconducting transitions, but have differences in the measured resistance, meaning the measured residual resistance is an artifact. Moreover, we use lock-in #1 to measure a pure Nb film (40 nm), and the residual resistance below the superconducting temperature is the same value as in the $K_{1-x}V_3Sb_5$ JJ (Fig. S1a). This further confirms that the residual resistance is an artifact from the lock-in, and the JJ does enter a complete superconducting.

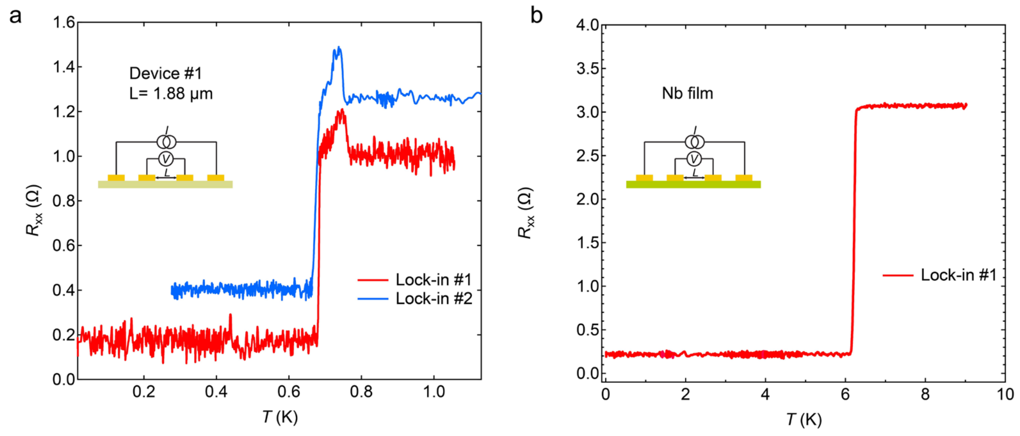

Figure S1. Residual of lock-in measurement. a, Resistance versus temperature (*R*-*T*) curves measured using two different lock-in amplifiers. The inset is the schematic measurement circuit. **b,** *R*-*T* curve of a Nb film measured by lock-in #1 using the same circuit as in (**a**).

S2. Superconductivity of $K_{1-x}V_3Sb_5$ Josephson Device #2

Figure S2 shows the superconducting property of two channels in Josephson Device #2. These two channels show slightly different critical temperatures, normal state resistances and critical currents. The Josephson junction with L = 0.1 μm has a smaller normal state resistance but larger critical current compared with the junction with L = 0.9 μm, as expected. However, the corresponding I_cR_N is close to each other, which is about 124 μV and 136.8 μV for L = 0.1 μm and L = 0.9 μm, respectively. This also indicates, as discussed in the main text and Device #1, that I_cR_N is dominated by the interface in our devices. The I_cR_N values in Device #2 is also close to the results in Device #1. The temperature dependence of critical current is characterized, as the result for L = 0.9 μm shown in Fig. S2c. The critical temperature gradually decreases with increasing temperature.

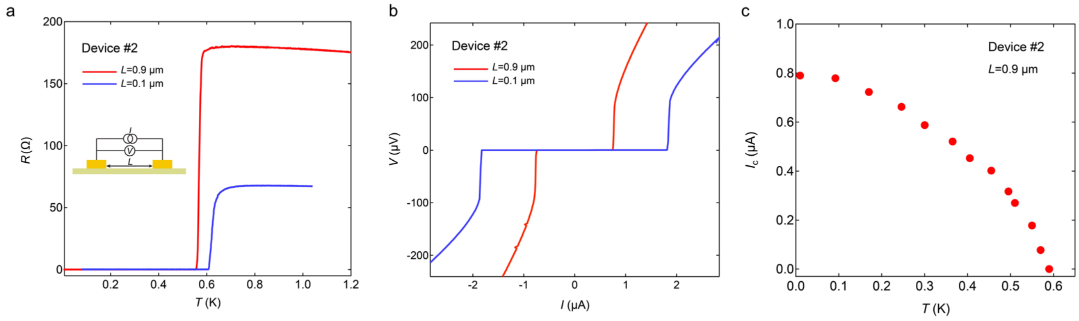

Figure S2. Superconductivity of Josephson Device #2. a. Temperature dependence of resistance in different Josephson channels measured by the two-probe method. **b.** Voltage vs current for Josephson junctions with different channel lengths measured at 20 mK. **c**. I_c vs T data (red dots).

S3. Transport data of $K_{1-x}V_3Sb_5$ devices with Au electrodes

KV_3Sb_5 devices with Au contacts (~ 60 nm) were fabricated to study the intrinsic properties at ultra-low temperature. We found that some of the devices show signs of superconductivity while others do not. Figure S3a and S3b shows the R vs T of a non-superconducting Au-contacted device (Device #3) with length (L), width (W) and thickness (t) of 10 µm, 5 µm, and 104 nm, respectively. No superconducting transition is observed. Note that the CDW transition and anomalous Hall effect still preserve in the non-superconducting samples, as shown in Fig. S3a and Fig. S3c. Figure S4, on the other hand, shows the transport properties of a superconducting device with $T_c \sim 0.65$ K and $I_c \sim 18$ µA. The R vs B curves show typical superconducting breaking without any signs of reversion for different field sweeping directions (Device #4, $L \approx 9.6$ µm, $W \approx 6.5$ µm, $t \approx 87$ nm). The critical field B_c is about ~20 mT for out-of-plane field and ~100 mT for in-plane field. In addition, the I_c gradually decays with increasing magnetic field, typical of a uniform superconductor.

To examine the origin of the sample variation of superconductivity in the KV_3Sb_5 devices, we used Energy Dispersive X-ray Spectroscopy (EDS) to characterize the composition of each device. All of the samples are potassium deficient, but devices showing intrinsic superconductivity are closer to the ideal 1:3:5 stoichiometry (*e.g.* Device #4 and #5) than the non-superconducting devices (Table S1). In addition, there is Nb impurity in the superconducting $K_{1-x}V_3Sb_5$ devices; the Nb is not expected since no source was included during the growth process but its presence may influence the superconductivity of, for example, Device #4. The variation between different devices is likely due to crystal homogeneity and may be addressable through synthesis refinement. This also indicates that the intrinsic

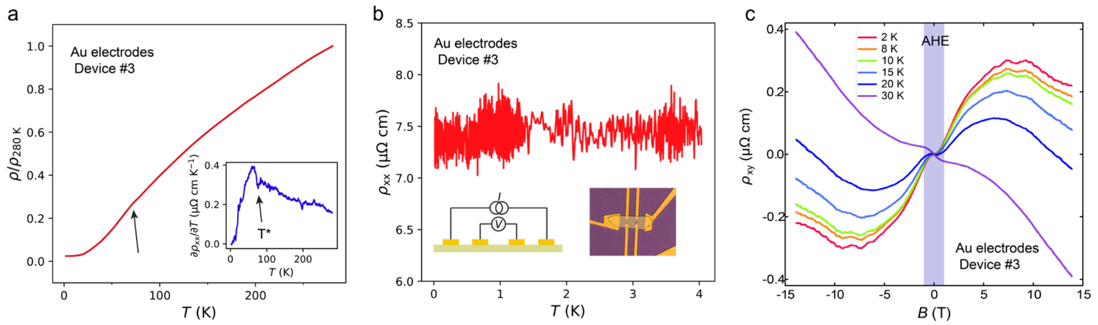

Figure S3. Properties of non-superconducting Device #3 with Au contacts. **a**. Relative temperature dependence of longitudinal resistivity, the inset is the differential longitudinal resistivity, the signal of CDW transition is marked by black arrows. **b.** Temperature dependence of longitudinal resistivity at low temperature, the insets are schematics of the measurement circuit (left) and devices image (right). **c**. Magnetic field dependence of Hall resistivity measured at different temperatures. The signal of anomalous Hall effect (AHE) is marked by purple region.

superconductivity of $K_{1-x}V_3Sb_5$ may be very sensitive to the composition, which is valuable to explore in future work. Note that the superconductivity of Device #5 is similar with Device #4, which possesses T_c ~ 0.6 K, I_c ~ 13 μA and B_c ~ 18 mT (out-of-plane field). The smaller T_c in $K_{1-x}V_3Sb_5$ than stoichiometric KV_3Sb_5 with T_c ~0.93 K (Ref. *15*) also indicate the weaker superconductivity in samples with deficiency. Note that, the $K_{1-x}V_3Sb_5$ flakes used in the fabrication of Josephson junction have large potassium deficiency, as shown in Table S1.

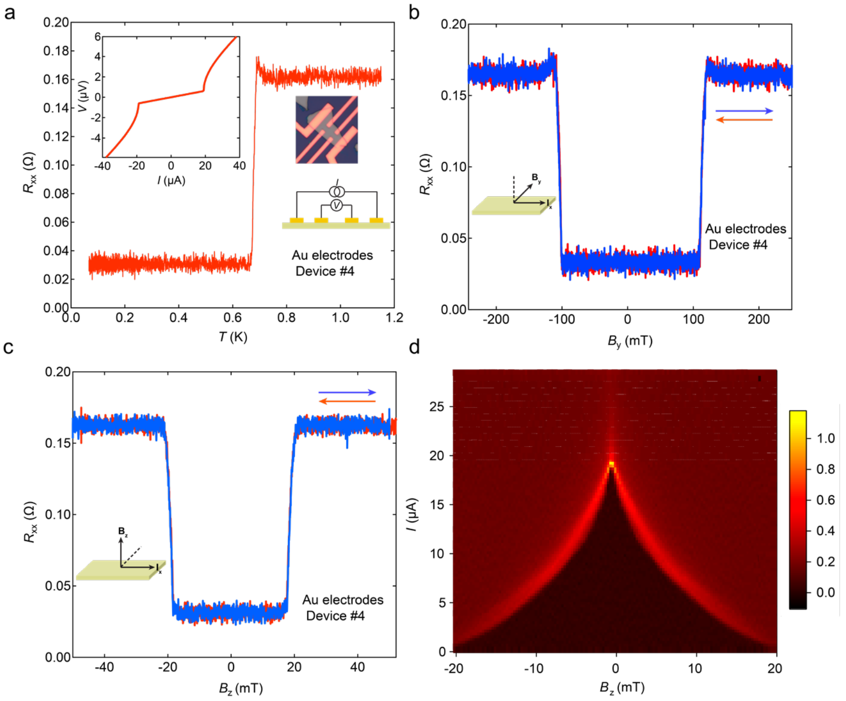

Figure S4. Superconductivity in KV_3Sb_5 Device #4 with Au contacts. a, Main panel: *R* vs *T* curve, the insets are V vs I curve measured at 20 mK, schematic measurement circuit, and device image. **b,** *R* vs *B* curve for up and down sweep of in-plane magnetic field. **c,** *R* vs *B* curve for up and down sweep of out-of-plane magnetic field. **d,** Field dependence of critical current of Device #4 with out-of-plane magnetic field.

Table S1: Stoichiometry in the channel of various $K_{1-x}V_3Sb_5$ devices characterized by EDS

	Contacts	x in ($K_xV_3Sb_5$)
Device #1 JJ (main text)	Nb	0.74(2)
Device #2 JJ	Nb	0.70(1)
Device #6 JJ	Nb	0.69(3)
Device #3<br>Not superconducting<br>(~1% Nb impurity)	Au	0.82(2)
Device #4<br>Superconducting<br>(~5% Nb impurity)	Au	0.87(2)
Device #5<br>Superconducting<br>(~1.5% Nb impurity)	Au	0.92(3)

S4. Discussion of proximity-induced superconductivity in Nb-$K_{1-x}V_3Sb_5$-Nb junction

As discussed in Section S3, $K_{1-x}V_3Sb_5$ can be intrinsically non-superconducting for high deficiency. The JJ devices in this paper are highly potassium deficient compared with the superconducting Au-contacted devices. In this section, we discuss the superconducting property of Nb-$K_{1-x}V_3Sb_5$-Nb in detail to show that the superconductivity in the junction is induced by proximity effect between Nb electrode, and exclude other possibilities such as the intrinsic superconductivity, and influence of inhomogeneous superconducting region.

First, the Nb-$K_{1-x}V_3Sb_5$-Nb junction in the main text (Device #1) has shown different critical temperature T_c (Fig. 1c) and critical current I_c (Fig. 2) of different channels made on one same $K_{1-x}V_3Sb_5$ flake with uniform width and thickness, this violates the normal behavior of an intrinsic superconductor with uniform width and thickness which should have constant T_c and I_c. In addition, the similar $I_c\underline{R}_n$ (R_n is normal state resistance of JJ) in the different channels of the Nb-$K_{1-x}V_3Sb_5$-Nb junction also indicates that the proximity effect induced by Nb contact determines the supercurrent of the junction (Fig. 2b).

Second, the magnetic field dependence of interference pattern shows the Fraunhofer-like pattern measured when magnetic field is applied in-plane further, which is a typical feature of Josephson effect that confirms the presence of the Josephson coupling. In addition, an important evidence that excludes intrinsic superconductivity is the larger critical field in Nb-$K_{1-x}V_3Sb_5$-Nb Josephson junction, where the critical fields are ~85 mT for out-of-plane field (B_z), and ~250 mT for in-plane field (B_y) (Fig. 3 in the main text). However, in the intrinsically superconducting Au-contacted $K_{1-x}V_3Sb_5$, the critical field is only ~15-20 mT for B_z, and ~100 mT for B_y (Fig. S4); much smaller than that in Nb-$K_{1-x}V_3Sb_5$-Nb junction. The larger critical field in Nb-$K_{1-x}V_3Sb_5$-Nb JJ and sustaining of the Fraunhofer pattern/fast oscillation up to the critical field, strongly indicate that the supercurrent in Nb-$K_{1-x}V_3Sb_5$-Nb junction is induced by the proximity effect.

Thirdly, the asymmetry and reversion of R vs B as well as the complex Josephson interference pattern (asymmetry and minimum center) are only observed in superconducting state of JJ. But in the intrinsic superconducting devices with Au contacts, there is no reversion of R vs B and no suppression of I_c at zero field (Fig. S4); clearly distinct from the JJ device behavior.

Based on these multiple evidences, we can conclude that the superconductivity observed in the main text are induced by proximity effect with Josephson coupling, and is not intrinsic.

Another question is that since it's hard to fully avoid inhomogeneity in the sample, it's necessary to consider whether the coupling between inhomogeneous superconducting region contribute to the Josephson coupling in the JJ devices. This can be excluded due to some reasons. First, there is no superconducting transition signal on the R vs T measurement in Au-contacted $K_{1-x}V_3Sb_5$ non-superconducting devices, it means that even if there is inhomogeneous, it should be quite small and doesn't affect the electrical signal, because if there were large inhomogeneous superconducting regions in the samples, there should have at least a partial signal of superconductivity on R vs T measurements (partial drops).

Secondly, the higher critical field in the $K_{1-x}V_3Sb_5$ JJ device (Device #1) than in the intrinsic superconducting samples (discussed above) also excludes the contribution of inhomogeneous superconducting region. Even if there was inhomogeneous doping, the intrinsic superconductivity is destroyed at high field and could not contribute to the Josephson relation through grains. And, as shown in Figure 3 and 4 in the main text, the fast oscillation (for B_z) and Fraunhofer pattern (for B_y) sustain to high field, which cannot be induced by the inhomogeneous superconductivity. In addition, the similar I_cR_n for different channel lengths up to 6.5 μm in Device #1 and similar value in $K_{1-x}V_3Sb_5$ JJ Device #2 (data shown in section S2 of SI), also indicates that the proximity effect is between the superconducting electrodes.

In addition, we specifically fabricated a junction of superconducting $K_{1-x}V_3Sb_5$ barrier (x~0.13) to check how superconducting barrier will influence the behavior of junction. Figure. S5a shows the device (Device #7), where both Au and Nb electrodes were fabricated to check the intrinsic property of the material and the proximity effect from Nb simultaneously. The intrinsic property of the sample is measured using Au electrodes, which is superconducting and the I_c vs B pattern shows the standard feature of a superconductor with a decreasing of I_c when increasing field (out-of-plane field B_z) and the

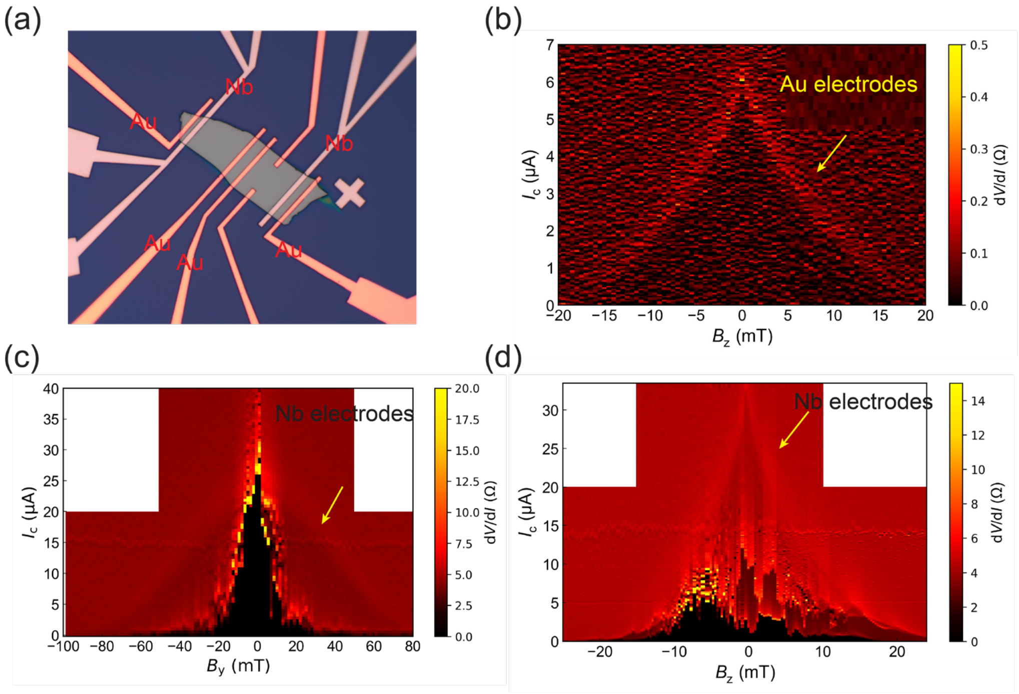

Figure S5. Results of Device #7 with superconducting $K_{1-x}V_3Sb_5$ (x~0.13) barrier. (a) Image of Device #7 with $K_{1-x}V_3Sb_5$ (x~0.13), Au and Nb electrodes are labeled. The length of scale bar is 10 µm. (b) I_c vs B vs dV/dI plot measured by Au electrodes for out-of-plane magnetic field, the field dependence of critical current is marked by yellow arrow. (c) I_c vs B vs dV/dI plot measured between Nb electrodes for in-plane magnetic field, the external weak peak is marked by yellow arrow. (d) I_c vs B vs dV/dI plot measured between Nb electrodes for out-of-plane magnetic field, the external weak peak is marked by yellow arrow.

maximum I_c is ~ 6 µA (Fig. S5b). Similarly, an I_c vs B pattern was measured between the Nb electrodes, which shows prominent features that are different with intrinsic superconductivity. The first one is an *external weak pattern* in the I_c vs B patterns for both B_y and B_z (marked by yellow arrows in Fig. S5c and S5d), which is very similar to the behavior of intrinsic superconductivity (I_c decreases with increasing B). However, the supercurrent (peak value of I_c~ 30µA) is much higher than intrinsic supercurrent (I_c ~6 µA) measured between the Au electrodes, indicating that they are not from intrinsic supercurrent, but should be influenced by the Nb electrodes. We suspect that this weak pattern might come from the enhanced superconductivity of $K_{1-x}V_3Sb_5$ by Nb electrode, more future works are needed to have a clear understanding of it.

The second feature is the anisotropic inner interference patterns. Although they are not of very good quality (possibly due to a large junction area and the irregular shape of the sample), there is a prominent difference for the out-of-plane and in-plane field measurements. The inner interference pattern has a central peak of I_c (maximum I_c~30 µA) and some oscillation signal when increasing in-plane magnetic field (Fig. S5c), however, the supercurrent is suppressed for the out-of-plane field (maximum supercurrent is only around ~10µA), as shown in Fig. S5d. These anisotropic inner interference patterns are very similar to the anisotropic features in the main text (Device #1). In addition, the I_cR_n (I_c~30 µA, two-probe R_n~4.2 Ω) is around 126µV, which is on the same order as the value in Device #1 in the main text.

These results indicate that there is superconducting coherence between Nb electrodes, which is influenced by the KV_3Sb_5 barrier (with time-reversal symmetry breaking and internal anisotropic magnetic moment), even in a long SS'S junction (30µm) with a superconducting $K_{1-x}V_3Sb_5$ barrier. This long coherence is indeed surprising, we expect future works of much longer junctions may be interesting to study when the coherence will disappear.

But importantly, different with the SS'S like junction, there is *no external weak pattern* in I_c vs B (like intrinsic SC) observed in the JJs of the highly-potassium deficient $K_{1-x}V_3Sb_5$ in the main text. This further proves that the device in the main text is not intrinsic superconductor, and as discussed before, the supercurrent also cannot come from the inhomogeneous superconducting region.

S5. Additional data for reversed *R* vs *B* curves in $K_{1-x}V_3Sb_5$ JJ

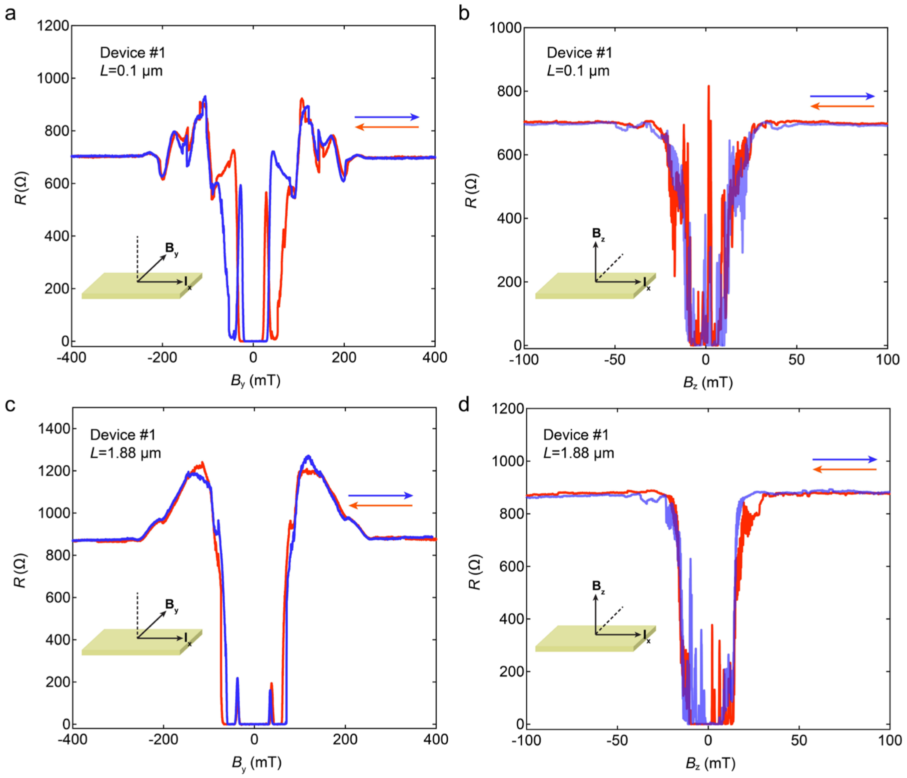

Figure S6. Reversed *R* vs *B* curves for other Josephson channels of Device #1. (**a-b**) and (**c-d**) are resistance vs field curves of Device #1 with L = 0.1 μm and L = 1.88 μm, respectively, which are measured by sweeping in-plane and out-of-plane magnetic field. The insets are schematic images of field directions. The up-sweep and down-sweep directions are indicated by black and red arrows. The *R*-*B* curve shows reserved feature between up-sweep and down-sweep in all the measured channels.

S6. Absence of reversion of R vs B curve in Nb-graphene-Nb JJ

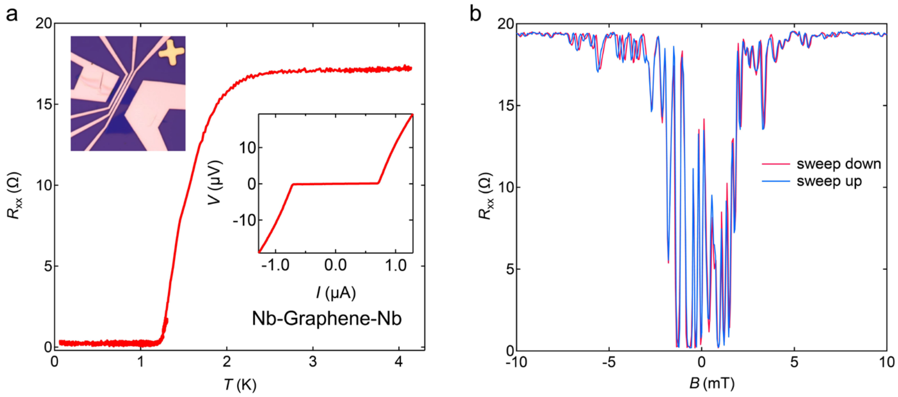

Figure S7. Property of Nb-graphene-Nb junction. (**a**) R vs T curve with $T_c \sim 1.5$ K of graphene JJ, the insets are V vs I curve and optical image of the device. (**b**) The R-B curve measured for out-of-plane magnetic field, the sweep up and sweep down curves are overlap with each other, without reversion.

S7. Interference pattern of Device #1 for up-sweep of magnetic field

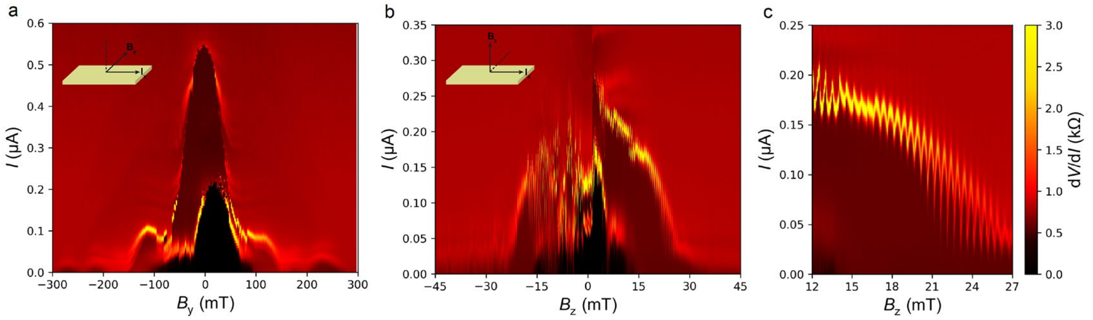

Figure S8. Interference pattern of Device #1 for applied magnetic field sweeping from negative to positive. a and **b** are color maps of dV/dI versus current and magnetic field measured in Device #1 at 20 mK with applying in-plane magnetic field and out-of-plane magnetic field, respectively. The field is changing from negative to positive during the measurement. The insets denote magnetic field and current directions. **c,** The enlarged plots of fast oscillation in (**b**) for applying out-of-plane field.

S8. Discussion of two superconducting channels in $K_{1-x}V_3Sb_5$ Josephson junction

In Figure 4 of main text, the interference patterns measured in Device #1 with L = 6.05 μm for both in-plane and out-of-plane fields show two sets of patterns with the outside pattern presenting prominent fast oscillations and dominating the main current-phase relations. At the same time, we found two transitions in the R-T curves in Josephson junction with long channel lengths, as shown in Fig. S9a. The junction with L = 6.05 μm presents an incomplete transition at T_1 = 0.87 K and a full superconducting transition at T_2 = 0.67 K. The R vs B curves for L = 6.05 μm measured at different temperatures also present a plateau, which has the same value of resistance with the plateau between T_1 and T_2 on R-T curve, as marked by the black arrows. The curves of different temperatures are shifted in Fig. S9b to make the feature of each curve clear. It is noticeable that the resistance can reach zero at small field when $T < T_2$, however, it can only reach the value of plateau when $T_2 < T < T_1$. These results indicate that there are likely two superconducting channels with different critical temperatures and fields in the long $K_{1-x}V_3Sb_5$ JJ.

It is difficult to clearly reveal the origin of these two superconducting channels. We suspect that one possibility might be related to the edge and bulk dominated states. In the L = 6.05 μm JJ, the fast

oscillation appears by 650 mK and survives above the plateau resistance (marked by black arrows) after applying magnetic field. This implies that the fast oscillation may be related to the first superconducting transition (T_1). As discussed in the Fig. 4 of main text, the fast oscillation strongly resembles the fingerprint of edge supercurrent and supported by theoretical expectation of edge states, the two transitions may be arising from the proximitized edge and bulk dominated states in $K_{1-x}V_3Sb_5$. Notably T_1 and T_2 merge together as the channel lengths decrease which is expected as the contribution of the edge state is more obvious in long channels. This phenomenon was seen in $MoTe_2$, where it is also reported that the edge superconductivity is easier to be observed in longer channels (Ref. (*65*)), consistent with our observations. More work is required to fully tease out and understand the underlying physical origin.

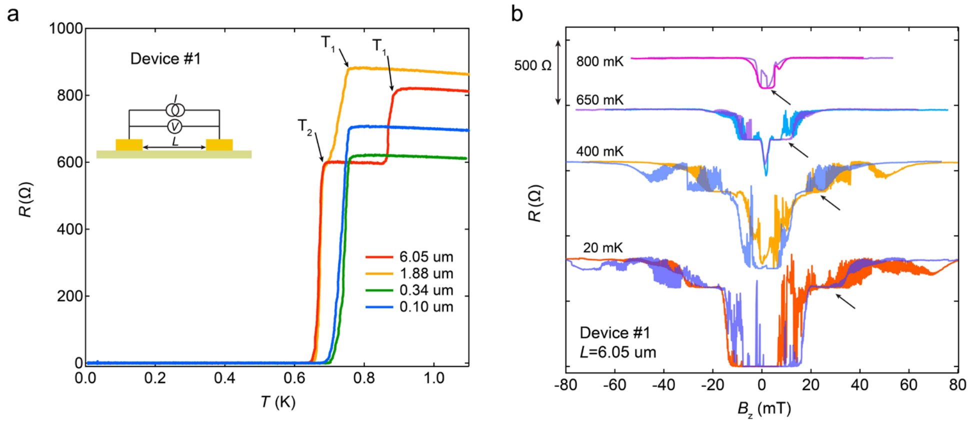

Figure S9. Two steps transition on *R*-*T* and *R* vs *B* curves. a, The temperature dependence of resistance for different channels in Device #1, the transition position is indicated by black arrows. The inset is the schematic measurement circuit. **b,** Resistance vs out-of-plane field at different temperatures measured in Device #1 with L = 6.05 μm, the R vs B curves for each temperature include both up-sweep and down-sweep. To clearly show the features on the R vs B curves, the R vs B curves for different temperatures have been shifted.

S9. Analysis of current density distribution

In a Josephson junction, the phase relation of the maximum critical current $I_c^{max}(B)$ is determined by the distribution of current density along the direction perpendicular to both the applied magnetic field and current direction. Assuming a Josephson junction with a length L in the x direction, a fixed magnetic field applied along the y direction will be related to a complex critical current function, $J_C(\beta)$, described by

$$J_C(\beta) = \int_{-\infty}^{\infty} d\, z\, J_S(z) e^{i\beta z} \quad (1)$$

Where $J_S(z)$ is the distribution of supercurrent along the z direction, the phase $\beta = 2\pi L_{eff} B/\Phi_0$, the effective junction length $L_{eff}=L+2L'$ (L' is the penetration depth of superconducting electrodes), and $\Phi_0 = h/2e$ is the magnetic flux quantum (Ref. (*39*)).

In experiment, the detected $I_c^{max}(\beta)$ is the absolute value of $J_C(\beta)$, *i.e.* $I_c^{max}(\beta) = |J_C(\beta)|$. According to the Fraunhofer-like pattern from the applied in-plane field, B_y with $B_y \perp I$, we can extract the supercurrent profile along the z direction in our device (perpendicular to the surface). To get the current profile of $J_S(z)$, the complex critical current function $J_C(\beta)$ must be recovered. According to Eq.1, $J_C(\beta)$ can be separated into two parts:

$$J_C(\beta) = I_E(\beta) + iI_O(\beta) \quad (2)$$

$$I_E(\beta) = \int_{-\infty}^{\infty} d\, z\, J_S(z) \cos(\beta z) \quad (3)$$

$$I_O(B) = \int_{-\infty}^{\infty} d\, z\, J_S(z)\, sin(\beta z) \quad (4)$$

The experimentally measured critical current $I_c^{max}(\beta) = \sqrt{I_E^2(\beta) + I_O^2(\beta)}$. It can be understood that $I_O(\beta)$ primarily influences the critical current at the minima, and $I_c^{max}(\beta)$ is determined by $I_E(\beta)$

away from the minima. One can recover $I_E(\beta)$ by multiplying a flipping function that alternates the sign across each minimum in the intereference pattern. Figure S10c is the extracted **even** part of critical current and the flipping function is shown in Fig. S10a (red curve). At the minima of $I_E(\beta)$, the **odd** part $I_O(\beta)$ determines the critical current, which can be obtained by interpolating between the minimum of $I_c^{max}(\beta)$ and the sign flip between each lobes, and the result is shown in Fig. S10b. After recovering the even and odd parts of $J_C(\beta)$, the distribution of critical current is obtained by

$$J_S(z) = \left|\frac{1}{2\pi}\int_{-t/2}^{t/2} d\,\beta\, J_C(\beta)e^{-i\beta z}\right| \tag{5}$$

where t is the thickness along z direction. Figure S10d shows the extracted current profile; the supercurrent is broadly distributed in a thin slab of the bulk approximately 5 nm thick.

We also extracted the current profile in the same way via inverse Fourier transform (IFT) for the I_c vs B curve for the out-of-plane field. The results are shown in Fig. S11. Unlike the uniform distribution of supercurrent contributed by only the bulk state found for in-plane-field, here there are two prominent peaks on the current profile, indicating the existence of edge supercurrent in the Josephson jucntion (Ref. (*39*)). It should be pointed that the critical current at the minima of each lobe is far from zero and the supercurrent near zero field is suppressed; these features may influence the quantitative accuracy of the IFT, but the two peaks qualitatively indicate the existence of edge suppercurrent.

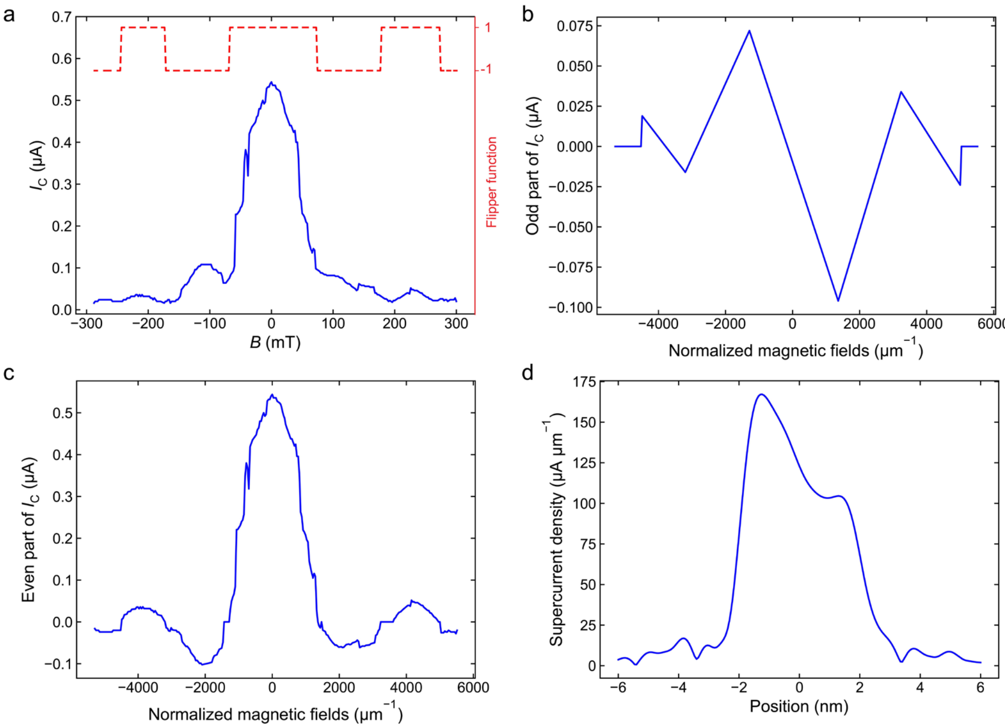

Figure S10. Current profile extraction. a, Interference pattern of the critical current $I_c^{max}(B)$ for the in-plane magnetic field B_y, the red curve is flipping function for extracting the even part of complex critical current. **b** and **c** are odd and even part of complex critical current, respectively. **d.** The extracted current density profile that corresponds to interference pattern in **a**.

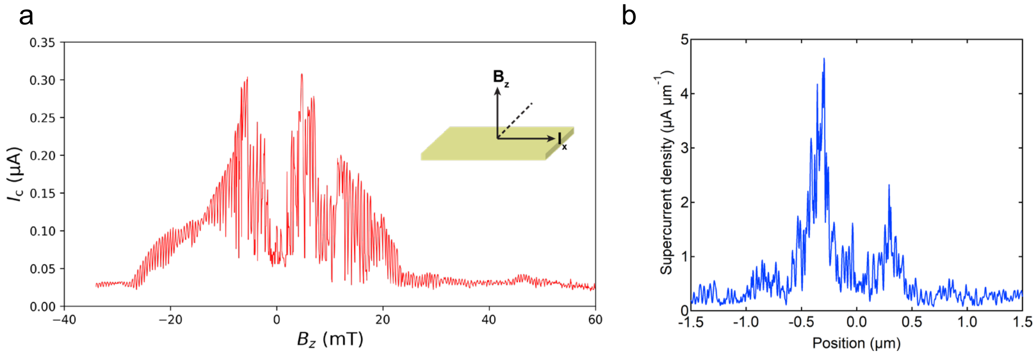

Figure S11. Current profile when applying out-of-plane magnetic field. a. I_c vs B curve, inset is schematic of magnetic field and current direction. **b.** The extracted current profile by performing inverse Fourier transform for I_c vs B curve in **a.**

S10. (001) Surface states and $\mathbb{Z}_2$ protected states in KV_3Sb_5

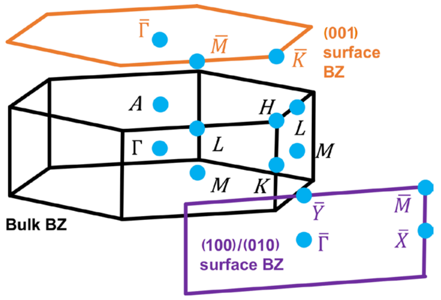

Figure S12| Bulk and surface hexagonal Brillouin zones. Here we provide labels for the high symmetry points used in our electronic structure calculations. The bulk Brillouin zone and high-symmetry points are labelled in black, while the (001) and (100)/(010) surface Brillouin zones are labelled in orange and purple, respectively. Note that surface projection can map one bulk BZ point to multiple surface points. Important for interpretation of Fig. S14—in which the $\mathbb{Z}_2$ states can be viewed near the $\bar{M}$ and $\bar{X}$ points on the (001) and (100)/(010) surfaces, respectively—is that these surface points are both projections of the bulk M point.

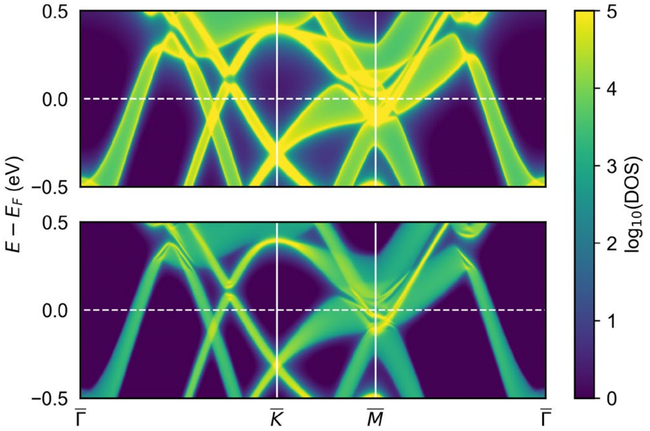

Figure S13| (001) surface states in KV_3Sb_5. In addition to the (100)/(010) edge states described in Fig. 4d in the main text, we provide here the (001) spectral density of states for the bulk (top panel) and surface

(bottom panel). Previous work Refs. (*14*) and (*18*) has shown that this calculated spectral density accurately recreates the experimentally measured (001) ARPES spectrum of both KV_3Sb_5 and CsV_3Sb_5 crystals. A number of bright surface bands can be viewed in the bottom panel which are not present in the bulk. $\mathbb{Z}_2$ surface states, previously characterized in-depth for CsV_3Sb_5 (Ref. (*14*)), are present just above the $\bar{M}$ point. Additional details on $\mathbb{Z}_2$ states in KV_3Sb_5 are provided in Fig. S14. Density of states units are unitcell^{-1} eV^{-1} Å^2.

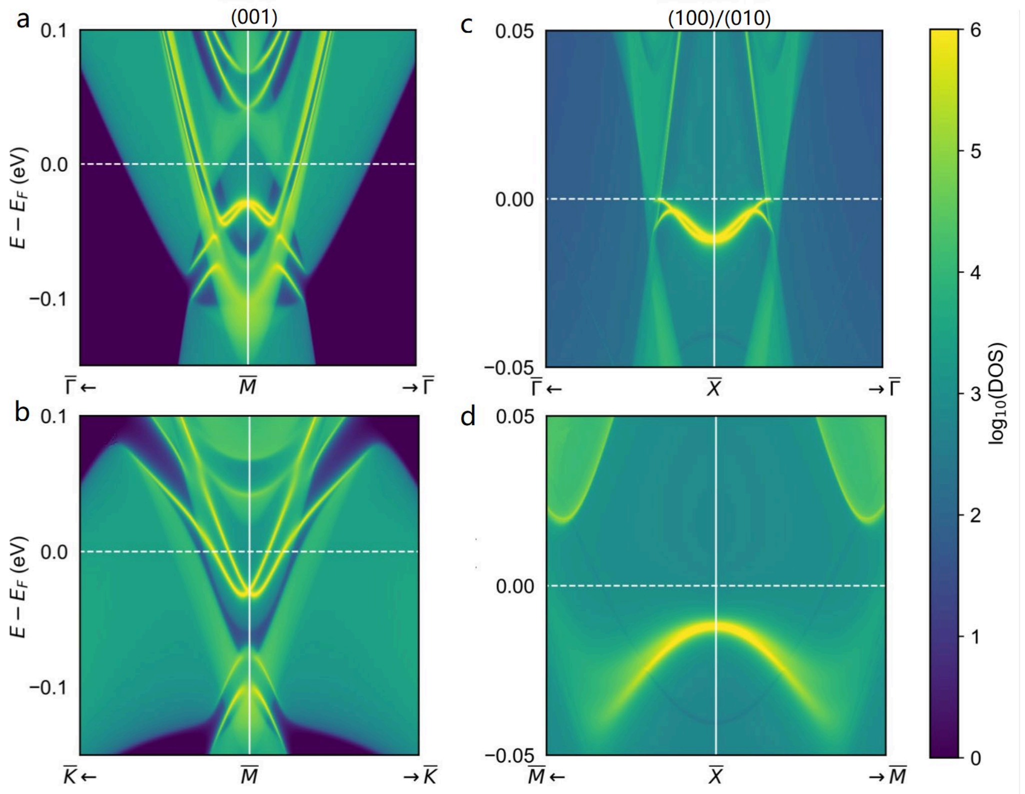

Figure S14| $\mathbb{Z}_2$ states in KV_3Sb_5. Here we provide close-up views of the $\mathbb{Z}_2$-protected surface Dirac crossing in KV_3Sb_5. The panels show these surface states along high symmetry lines not just for the (001) surface but also for the (100)/(010) surfaces which are more relevant to the edge conductivity experimentally uncovered in this work. (**a**-**b**) present the surface Dirac crossing at the (001) $\bar{M}$ point along the $\bar{\Gamma}$-$\bar{M}$-$\bar{\Gamma}$ and $\bar{K}$-$\bar{M}$-$\bar{K}$ lines. (**c**-**d**) present the surface Dirac crossing at the (100)/(010) $\bar{X}$ point along the $\bar{\Gamma}$-$\bar{X}$-$\bar{\Gamma}$ and $\bar{M}$-$\bar{X}$-$\bar{M}$ lines. These crossings can be viewed just below the Fermi level in Fig. 4d and Fig. S13. Note that the surface band dispersion is very sensitive to the choice of surface termination layer; this accounts for the small energy difference between the centers of the Dirac crossing on the two surfaces. Density of states units are unitcell^{-1} eV^{-1} Å^2.
